# The Serum Concentration of the Calcium Binding Protein S100B is Positively Associated with Cognitive Performance in Older Adults

**DOI:** 10.3389/fnagi.2013.00061

**Published:** 2013-09-30

**Authors:** Virginie Lam, Matthew A. Albrecht, Ryusuke Takechi, Corey Giles, Anthony P. James, Jonathan K. Foster, John C. L. Mamo

**Affiliations:** ^1^School of Public Health, Curtin Health Innovation Research Institute, Biosciences Research Precinct, Faculty of Health Sciences, Curtin University, Perth, WA, Australia; ^2^School of Psychology and Speech Pathology, Curtin Health Innovation Research Institute, Faculty of Health Sciences, Curtin University, Perth, WA, Australia; ^3^Neurosciences Unit, Health Department of Western Australia, Perth, WA, Australia

**Keywords:** S100B, neuropsychological measures, Alzheimer’s disease, Bayesian analysis, neuro-inflammation

## Abstract

S100B is a calcium binding peptide produced predominantly by astroglial cells in the central nervous system. S100B paradoxically has neurotrophic and apoptotic effects, dependent on extracellular concentration. This study investigated the relationship between serum S100B levels and neuropsychological performance across a range of cognitive domains in healthy older aged adults. A cohort of 219 participants between the ages of 43 and 84 years (141 female) were recruited. Subjects provided a fasting blood sample for S100B measurement (Mean = 0.24 ng/mL, SD = 0.14) and completed a battery of neuropsychological tests. S100B concentrations (both with and without the covariates of age and sex) were positively associated with the following measures of cognitive performance: digit-symbol coding, Stroop test, and measures of verbal ability. The results from this study show that serum S100B is positively associated with better cognitive performance in healthy older adults.

## Introduction

In 1965, Moore described a mixture of low molecular weight proteins belonging to the calcium-sensor-binding-proteins super-family (Moore, 1965). Indicative of their solubility in saturated ammonium sulfate solution, S100A is a heterodimer synthesized in muscles and neurons, whereas S100B is homodimer produced by neural astroglial and Schwann cells (Heizmann, 2002). The biological effects of S100 proteins are functional following binding of ionized calcium, which induces a conformational change and exposure of functional hydrophobic residues (Smith et al., 1996).

Key functional intracellular effects of S100B are purported to be in regulation of cell proliferation and cytoskeletal structure (Sorci et al., 1998). Extracellular effects at nanomolar concentrations of S100B have potent neurotrophic and gliotrophic actions, principally by ameliorating the effects of altered redox states that occur as a consequence of mitochondrial dysfunction (Selinfreund et al., 1991; Donato, 2001). In addition to its significance in central nervous system (CNS) development, S100B may be restorative following brain injury (Ellis et al., 2007). However, paradoxical biological effects of S100B are reported at micromolar levels in extracellular fluids such as plasma or cerebrospinal fluid (CSF). *In vitro* studies have identified that at these higher concentrations extracellular S100B binds to the receptor for advanced glycation end products, stimulating the caspase pathway (a pivotal mediator for programed cell death, necrosis, and inflammation) (Huttunen et al., 1999). At these concentrations, cytokine production is enhanced resulting in increased production of potentially cytotoxic amounts of reactive oxygen species and nitric oxide (Bianchi et al., 2007). It is these properties of S100B that have led to the hypothesis that elevated S100B contributes to neurovascular inflammatory disorders.

The demonstrated paracrine and autocrine effects of S100B on neurons and glia and the neural-to-plasma kinetic gradient of S100B has raised the possibility that S100B could be a useful blood biomarker for disorders of the CNS. The relatively short half-life of plasma S100B (∼30 min) and renal clearance (2 h) would support the contention that a chronic change in serum S100B concentration may reflect homeostatic alterations of neural integrity (Jonsson et al., 2000). Yardan et al. (2011) provide an elegant review of S100B in the context of its putative function in individuals who have experienced head trauma, neurovascular degenerative conditions, or psychological disorders.

Alzheimer’s disease (AD), the most common neurodegenerative disorder, is clinically characterized by a progressive loss of cognitive functioning that typically starts with a decline in episodic memory (Backman et al., 2004). In AD and in subjects with frontotemporal lobe dementia, S100B levels were reported to be significantly increased compared to healthy controls (Green et al., 1997; Peskind et al., 2001). However, Yardan et al. (2011) suggested that S100B’s abundance and distribution and its putative role in the onset and progression of AD could notionally change over the time course of the disease. Chronically suppressed levels of extracellular S100B may be detrimental to neuronal function and be implicated in AD onset. However, during periods of heightened inflammation or more active plaque formation, greater S100B concentration in serum might be expected. The latter could be considered an injury response that provides benefit (at lower concentration) or exacerbates inflammatory sequelae (at higher concentrations). Thereafter, at the end-stages of AD, normal or perhaps decreased synthesis and secretion of S100B may occur. This was reported by Peskind et al. (2001) who found that S100B concentrations were increased in mild-to-moderate AD subjects but not in the advanced stage of the disease. Consistent with a transient functional role of S100B in neurodegenerative disease, Schaf et al. (2005) reported a correlation of S100B with the Hoehn and Yahr stage of Parkinson’s disease, but no difference *per se* when S100B was measured in Parkinson’s disease subjects versus controls. Furthermore, Nooijen et al. (1997) found no significant difference in concentration of S100B in various types of dementia apart from in subjects with Creutzfeldt–Jakob disease. However, an association with disease onset or progression was not considered in this study.

Cognition (as per complex biological phenomena in general) is a multifactorial entity that reflects the composite domains of perceptual speed, primary memory, secondary memory, verbal ability, linguistic abilities, and executive functioning. In subjects with mild-cognitive impairment, secondary memory (episodic memory) deficits and (to a lesser extent) perceptual speed and executive functioning deficits appear to be most indicative of subjects who will progress to AD (Weintraub et al., 2012). The fractionation of different elements of cognition assessed against biomarkers could provide a powerful approach to modeling the putative relationship(s) between cognitive outcomes and regulatory biological factors in the aging process (specifically concerning biological risk factors for pro-dromal AD). In this study, we therefore used a battery of tests that specifically evaluated each of the aforementioned cognitive domains, and we explored if there was a statistical association between cognitive test performance and serum S100B concentration. The hypothesis was that, in generally healthy subjects, the neurotrophic properties of S100B would generally correlate positively with cognitive capacity.

## Materials and Methods

### Participants

The study was approved by the Curtin University Human Research Ethics Committee (HR97/2011). A total of 250 participants (96 males, 154 females) over the age of 40 (range = 43–84 years) were recruited. All participants provided written consent and completed a medical history and medications questionnaire and were interviewed to confirm the information provided. Exclusion criteria for the study were: major surgery or a clinical event within 6 months; current diagnosis with a psychiatric disorder or taking psychotropic medications; hemophilia; cancer/chemotherapy; head injury within 5 years; diagnosis with HIV. Furthermore, participants were excluded from the statistical analyses if any of the following obtained: renal impairment; liver dysfunction; Mini Mental State Examination (MMSE) score <24.

### Serum S100B

Peripheral venous samples were collected into serum separator Vacutainer™ tubes (Becton Dickinson, Franklin Lakes, NJ, USA) following an overnight fast for at least 8 h. Samples were allowed to clot for 30 min and serum was isolated and stored at −80°C following low-speed centrifugation. Serum S100B was measured using a commercially available ELISA kit (Cosmo Bio, Japan) with an interassay coefficient of variance of 4.82–9.20%. The sensitivity and dynamic range of the S100B assay is 0.078–5 ng/mL (7–470 pmol/L).

### Neuropsychological measures

The cognitive tests were administered by trained staff under supervision of a registered clinical neuropsychologist. Tests were chosen based upon their widespread use, reliability, and validity, and to cover the principal domains of cognitive performance affected in age-related cognitive decline. The performance tasks included the MMSE (Folstein et al., 1975), Rey Auditory Verbal Learning Test (RAVLT) (Lezak et al., 2004), Delis–Kaplan Executive Function System (D–KEFS) verbal fluency subtests (Delis et al., 2001), 60-item Boston Naming Test (BNT) (Saxton et al., 2000), National Adult Reading Test (NART) (Nelson and Willison, 1991), Digit Span and Digit-Symbol Coding subtests from the Wechsler Adult Intelligence Scale-Third edition (WAIS-III) (Wechsler and Scale, 1997; Strauss et al., 2006), and the Stroop test (Victoria version) (Strauss et al., 2006).

### Statistical analysis

The relationship between serum S100B and cognitive performance was considered using a nested domain Bayesian mixed-model (Thurston et al., 2009). The nested domain model increases power by pooling outcome estimates from within a cognitive domain toward each other, reducing Type S (sign) and Type M (magnitude) errors through shrinkage toward common estimates (Gelman et al., 2012).

The principal domains assessed were D1 – verbal ability [BNT and D–KEFS fluency (letter fluency, category fluency, and category switching)]; D2 – Stroop [Dots, Words, and Colors response time, and interference (Colors/Dots) ratio]; D3 – secondary memory [total items recalled across learning trial, items recalled from interference list, short delay free recall, long delay free recall, recognition “hits,” short delay forgetting score (learning trial 5 – short delay), and long delay forgetting score (learning trial 5 – long delay)]; D4 – primary memory (digits forward and digits backwards); and D5 – perceptual speed (digit-symbol coding). The outcome measurements to be nested within each domain were chosen *a priori*. An objective Bayesian approach to setting the priors was used, that is all priors could be described as being weakly informative, or uninformative for the scale of the data. Priors for the overarching coefficients were described by a normal distribution with a mean = 0 and SD = 100. The remaining coefficients for the outcomes and domains were described as being derived from a normal distribution centered on 0 and a SD estimated from a half-Cauchy distribution centered on 0, and scale set to 25 (Gelman, 2006). Outcome level errors were modeled as being derived from a *t*-distribution to render the analysis robust (Lange et al., 1989; Kruschke, 2012). The prior for the SD for each outcome was described by a uniform distribution between 0 and 100 and the degrees of freedom parameter was estimated from the inverse of a uniform distribution with lower and upper limits of 0.001 and 0.5. A large estimate for the degrees of freedom parameter indicates that the residuals can be described by normal distribution, while a smaller degrees of freedom parameter indicates that the data have fatter tails and data points in this region are appropriately down-weighted. Each variable was scaled to a mean of 0 and a SD of 1. If a smaller score on any neuropsychological measure indicated “better” performance, the score was inverted. After 5000 adaptation steps and 50,000 burn-in steps, a total of 50,000 Markov Chain Monte Carlo (MCMC) samples (thinned every tenth step) were saved across three chains for the final parameter estimates. Convergence was confirmed by examining plots of the posterior and using the Gelman–Rubin diagnostic (Gelman and Rubin, 1992). All posterior distributions used for inference had a minimal effective sample size of at least 1000 (usually ∼10,000). The means ±95% highest density intervals (HDI) of the posterior distribution were used to describe the credibility interval for each of the parameter estimates (Kruschke, 2012). All statistical analyses were conducted in R version 3.0.0 using the “rjags” package (Plummer, 2013).

## Results

### Relationship between S100B and covariates age and sex

A total of 219 participants met the key inclusion criteria for this study. The mean age of the cohort was ∼65 years. All included participants had an MMSE scores >25. The mean serum S100B concentration was 0.24 ng/mL and normally distributed for the cohort; however, the range was substantial (Table 1). The covariates of age (slope = −0.001 ng/mL/year, HDI = −0.004, 0.002) and sex (contrast F-M = −0.02 ng/mL, HDI = −0.06, 0.02) were not substantially associated with S100B. By contrast, NART errors were negatively associated with S100B concentrations (slope = −0.004 ng/mL/error, 95% HDI = −0.007, −0.0008).

**Table 1:** **Measures of age, S100B concentration and MMSE scores and neuropsychological measures for the study cohort of 219 participants**.

		Mean	SD	Range
Sex (F | M)	141 | 78			
Age		64.9	7.3	43.6–84.2
S100B (ng/mL)		0.24	0.14	0.08–0.62
MMSE		28.7	1.3	25–30
NART error score		14.1	6.6	3–39
**NEUROPSYCHOLOGICAL MEASURES**				
**Perceptual speed**
Digit-symbol coding		64.6	13.9	23–95
**Primary memory**
Digits forward		6.8	2.0	6–16
Digits backward		10.4	2.2	3–18
**Secondary memory**
RAVLT sum of trials 1–5		44.5	9.6	16–64
RAVLT interference list		5.1	1.9	1–11
RAVLT short delay recall		9.1	3.1	0–15
RAVLT long delay recall		9.1	3.1	0–15
RAVLT recognition ‘Hits’		13.4	1.9	0–15
RAVLT decay T5 – short delay		2.1	2.0	−3–11
RAVLT ‘decay’ T7 – short delay		2.2	2.0	−3–8
**Stroop**
Stroop words time		18.5	7.3	11–101
Stroop dots time		13.9	3.5	7–36
Stroop colors time		29.9	10.1	14–80
Stroop interference (C/D)		2.2	0.66	0.8–4.8
**Verbal ability**
D–KEFS letter fluency		40.9	11.8	7–71
D–KEFS category fluency		44.5	8.9	23–69
D–KEFS switching total		13.6	2.9	4–21
Boston naming test		56.3	4.2	26–60

### Relationship between S100B and neuropsychological performance

**Figure 1:**
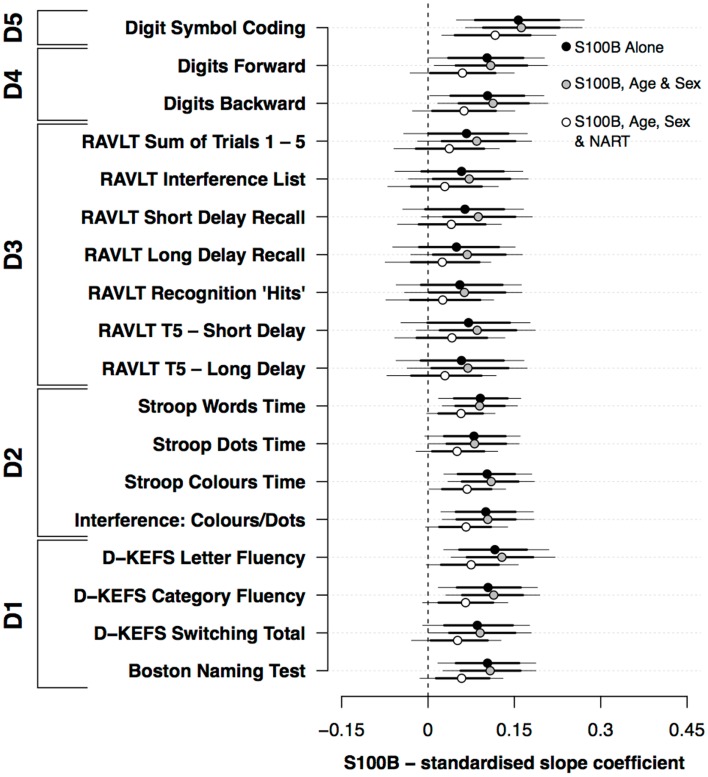
**Standardized slope coefficients (mean ±80%, 95% HDI) for the association between S100B and neuropsychological performance; both with and without the covariates of age, sex, and premorbid functioning**.

Figure 1 presents the standardized slope coefficients (mean ±80%, 95% HDI) for the association between S100B and neuropsychological performance both with and without the covariates of age, sex, and NART. S100B both with and without the covariates of age and sex was positively associated with performance on a range of neuropsychological measures. In the models that excluded covariates, S100B was positively associated with perceptual speed (D5), Stroop (D2), and verbal ability (D1). While the credible intervals for the primary memory (D4) and secondary memory (D3) domains included 0, their mean effect sizes were similar to the Stroop (D2) and verbal ability (D1) domains. Including the covariates age and sex did not appreciably alter the relationship between S100B and cognitive performance. By contrast, the addition of NART scores modestly reduced the mean estimates and associated confidence intervals across the range of neuropsychological measures used.

## Discussion

Serum S100B has been suggested to be a candidate marker of CNS injury because its concentration is increased after head trauma and in several neurodegenerative conditions (Sen and Belli, 2007). In these circumstances, heightened overproduction of S100B may amplify inflammatory processes and exacerbate cell stress as a consequence of altered redox state. An alternative interpretation is that elevated serum S100B is a consequence of amplified inflammation and cell stress, as several lines of evidence show significant positive trophic effects of S100B consistent with a neuroprotective function. This study explored for the first time the relationship between serum S100B and neuropsychological test performance across a range of cognitive domains in a relatively health cohort of older aged participants. Our analyses indicated that this study cohort manifested serum S100B concentrations which have been reported to support cell function and integrity in cell culture studies (i.e., <nanomolar range). No participant had serum S100B concentrations considered potentially indicative of CNS injury (i.e., micromolar range). Utilizing a nested cognitive domain model to explore the hypothesis of an association between cognitive capacity and serum S100B, the data indicates a positive association with all cognitive domains and in particular for perceptual speed, Stroop, and verbal ability. Adjustments for NART, but not age and gender, had a modest attenuating effect on the associations observed.

The largest effect size with respect to the association between cognitive performance and S100B was on the digit-symbol coding measure, reflecting perceptual, and motor speed. Similarly, performances on other speeded tasks (Stroop and verbal ability) were positively associated with serum S100B. The finding may indicate a beneficial association between S100B and “on line” speeded tasks. This is a potentially important finding, as “speed” has been considered a fundamental component of age-related CNS functional integrity (Salthouse, 1996). However, given that there are only very modest differences between the mean parameter estimates for the different cognitive domains that were evaluated in this study, the apparent selectivity of S100B for the speeded tasks may be more reflective of the psychometric properties of the tasks. For example, many of the secondary memory measures (which are derived from the RAVLT) only have a range of 15 discrete possibility outcomes, compared to the more continuous nature of the Stroop test and the larger range of possible scores on the Digit-Symbol Coding test. Therefore, an alternative hypothesis to be explored is whether S100B is selectively associated with particular cognitive domain(s) (for example, if the functional capacity of specific brain regions are associated with levels of S100B), or whether these concentrations are associated with a global enhancement of cognitive performance (as might be expected if cognitive processing were generally “faster” when higher concentrations of S100B are present within the healthy physiological range).

There has been significant interest in S100B as a potential marker of CNS trauma, distress, or pathological disturbances, and in this context S100B is described as an acute-phase response protein (Sen and Belli, 2007). In contrast, non-injurious and chronic levels of serum S100B would presumably represent constitutive rates of biosynthesis and metabolism. For this study, participants were currently functioning within the “healthy” range and individuals with head trauma, or other conditions known to significantly influence serum S100B concentration were excluded. Therefore, it appears reasonable to assume that S100B was not indexing CNS dysfunction.

Several lines of evidence support an important role for S100B in CNS development. Heightened S100B in response to stressors may be indicative of insult severity, although in many instances it has been suggested to be causally related to pathological sequelae. Intraventricular infusion of low concentrations of S100B induces neurogenesis within the hippocampus in a traumatic brain injury model, and this was associated with enhanced cognitive function (Kleindienst et al., 2005).

S100B is mainly found in astroglial and Schwann cells and is enriched in CSF relative to blood. Many studies have therefore suggested that elevated serum S100B could be a useful surrogate marker of blood-brain leakage (Marchi et al., 2003); indeed, this proposal is supported in clinical and animal head trauma findings. However, other sources of serum S100B could include adipocytes, chondrocytes, lymphocytes, bone marrow cells, and melanocytes, with clearance occurring predominantly via renal excretion. Based on the exclusion criteria indicated in this study (no recent head trauma, frank neurological disorder, or renal dysfunction), serum S100B homeostasis was evaluated in a “healthy” physiological context rather than in a neuropathological context, with the evidence suggesting positive associations with cognition in healthy participants.

The cross-sectional study design that was used unfortunately does not permit delineation of possible casual mechanisms with respect to the association between serum S100B and cognitive performance. Possible effects of S100B relevant to cognition may include improved redox state and cell function; suppression of neurovascular inflammation; enhanced conduction and transmission of nerve impulses (putatively of marked relevance given the findings on “speeded” cognitive tasks noted in this study); promotion of cell growth and/or differentiation or enhanced cytoskeletal structure.

Future studies to investigate the relationship of serum S100B levels and cognitive/neurological functions under stress/pathological conditions may be particularly warranted. The body of evidence to date suggests that S100B may be an acute-phase response-to-injury protein that confers positive trophic and functional effects of the neurovascular unit. However, S100B is not normally considered as a chronic modulator of neurovascular function. Evidence consistent with the latter may provide therapeutic opportunities particularly in the pre- and pro-dromal phase of neurodegenerative conditions such as AD. Realization of the clinical translatability will however require a comprehensive understanding of S100B metabolism, kinetics, and molecular mechanisms. Common to these proposed functional effects is binding of S100B to ionized calcium, which as a consequence exposes hydrophobic residues (thereby enabling S100B to interact with other proteins and thus exert a biological effect). Possible synergistic effects of S100B and calcium metabolism on cognition may therefore be of particular interest in future studies.

## Authors Contribution

Virginie Lam and John C. L. Mamo researched literature and conceived the study. Virginie Lam, John C. L. Mamo, Ryusuke Takechi, Corey Giles, Anthony P. James, and Jonathan K. Foster were involved in protocol development, gaining ethical approval, and patient recruitment. Virginie Lam, Matthew A. Albrecht, Jonathan K. Foster, and John C. L. Mamo were responsible for data and statistical analysis. All authors reviewed and edited the manuscript and approved the final version of the manuscript.

## Conflict of Interest Statement

The authors declare that the research was conducted in the absence of any commercial or financial relationships that could be construed as a potential conflict of interest.
